# Radiation-induced lung injury after breast cancer treatment: incidence in the CANTO-RT cohort and associated clinical and dosimetric risk factors

**DOI:** 10.3389/fonc.2023.1199043

**Published:** 2023-06-29

**Authors:** Anna Gueiderikh, Thomas Sarrade, Youlia Kirova, Brigitte De La Lande, Florent De Vathaire, Guillaume Auzac, Anne Laure Martin, Sibille Everhard, Nicolas Meillan, Celine Bourgier, Ahmed Benyoucef, Thomas Lacornerie, David Pasquier, Séverine Racadot, Alexandra Moignier, François Paris, Fabrice André, Eric Deutsch, Boris Duchemann, Rodrigue Setcheou Allodji, Sofia Rivera

**Affiliations:** ^1^ Gustave Roussy, Radiation Therapy Department, Villejuif, France; ^2^ Tenon Hospital, Radiotherapy Department, Paris, France; ^3^ Department of Radiation Oncology, Institut Curie, Paris, France; ^4^ University Versailles, St. Quentin, France; ^5^ Department of Radiation Oncology, Institut Curie, Saint Cloud, France; ^6^ Gustave Roussy, Comprehensive Cancer Research Center, Villejuif, France; ^7^ Centre for Research in Epidemiology and Population Health, U1018 Institut National de la Santé et de la Recherche Médicale (INSERM), Villejuif, France; ^8^ Paris-Saclay University, Unité Mixte de Recherche (UMR) 1018, Villejuif, France; ^9^ UNICANCER, Data Department, Kremlin Bicêtre, France; ^10^ Paris-Saclay University, Gustave Roussy, Institut National de la Santé et de la Recherche Médicale (INSERM) 1030, Villejuif, France; ^11^ Radiation Therapy Department, CH Victor Dupouy, Argenteuil, France; ^12^ Montpellier University, Montpellier, France; ^13^ Institut de Recherche en Cancérologie de Montpellier (IRCM), Institut National de la Santé et de la Recherche Médicale (INSERM) U1194, Montpellier, France; ^14^ Fédération Universitaire d’Oncologie Radiothérapie d’Occitanie Méditerranée, Institut régional du Cancer Montpellier (ICM), Montpellier, France; ^15^ Radiation Therapy Department, Henri Becquerel Center, Rouen, France; ^16^ Centre Oscar Lambret, Academic Department of Radiation Oncology, 3 rue Combemale, Lille, France; ^17^ Univ. Lille, &, Centre National de la Recherche Scientifique (CNRS), Centrale Lille, Unité Mixte de Recherche (UMR) 9189 – Centre de Recherche en Informatique, Signal et Automatique de Lille (CRIStAL), Lille, France; ^18^ Radiotherapy Department, Centre Léon Bérard, Lyon, France; ^19^ Radiotherapy Department, Institut de Cancérologie de l’Ouest, Nantes, France; ^20^ Nantes Université, Nantes - Angers Cancer and Immunology Research Center (CRCI2NA), Institut National de la Santé et de la Recherche Médicale (INSERM), Centre National de la Recherche Scientifique (CNRS), Nantes, France; ^21^ Gustave Roussy, Medical Oncology Department, Villejuif, France; ^22^ Paris-Saclay University, Gustave Roussy, Institut National de la Santé et de la Recherche Médicale (INSERM) U981, Villejuif, France; ^23^ Avicenne Hospital, Thoracic Oncology, Bobigny, France

**Keywords:** radio-induced lung injury, lug fibrosis, breast cancer, radiation therapy, CANTO cohort, RILI, dosimetric analyses

## Abstract

**Purpose:**

Radiation-induced lung injury (RILI) is strongly associated with various clinical conditions and dosimetric parameters. Former studies have led to reducing radiotherapy (RT) doses to the lung and have favored the discontinuation of tamoxifen during RT. However, the monocentric design and variability of dosimetric parameters chosen have limited further improvement. The aim of our study was to assess the incidence of RILI in current practice and to determine clinical and dosimetric risk factors associated with RILI occurrence.

**Material and methods:**

Data from 3 out of the 10 top recruiting centers in CANTO-RT, a subset of the CANTO prospective longitudinal cohort (NCT01993498), were retrospectively analyzed for RILI occurrence. This cohort, which recruited invasive cT0-3 cN0-3 M0 breast cancer patients from 2012 to 2018, prospectively recorded the occurrence of adverse events by questionnaires and medical visits at the end of, and up to 60 months after treatment. RILI adverse events were defined in all patients by the association of clinical symptoms and compatible medical imaging.

**Results:**

RILI was found in 38/1565 (2.4%) patients. Grade II RILI represented 15/38 events (39%) and grade III or IV 2/38 events (6%). There were no grade V events. The most frequently used technique for treatment was 3D conformational RT (96%). In univariable analyses, we confirmed the association of RILI occurrence with pulmonary medical history, absence of cardiovascular disease medical history, high pT and pN, chemotherapy use, nodal RT. All dosimetric parameters were highly correlated and had close predictive value. In the multivariable analysis adjusted for chemotherapy use and nodal involvement, pulmonary medical history (OR=3.05, p<0.01) and high V30 Gy (OR=1.06, p=0.04) remained statistically significant risk factors for RILI occurrence. V30 Gy >15% was significantly associated with RILI occurrence in a multivariable analysis (OR=3.07, p=0.03).

**Conclusion:**

Our study confirms the pulmonary safety of breast 3D RT in CANTO-RT. Further analyses with modern radiation therapy techniques such as IMRT are needed. Our results argue in favor of a dose constraint to the ipsilateral lung using V30 Gy not exceeding 15%, especially in patients presenting pulmonary medical history. Pulmonary disease records should be taken into account for RT planning.

## Introduction

Radiation-induced lung injury (RILI) is one of the most common clinically challenging toxicities induced after breast radiation therapy (RT). RILI encompasses two phases: an acute phase termed Radiation Pneumonitis, occurring within the first 6 months after RT, consisting in a lung inflammation phase responsible for dyspnea, cough, sometimes pleural effusion and a late phase called radiation lung fibrosis, leading to irreversible tissue damage (1, 2). In both acute and late phases and for all grades, RILI translates in radiologic alterations.

The guidelines parameters for measuring and reporting radiation toxicity and prevent the risk of RILI include the volume of lungs receiving a certain dose of radiation (Vx Gy) and the mean radiation dose (Dmean) to the lungs (3). RILI is one of the main toxicity risk factors that influence the technical RT planning of breast cancer treatment. RILI incidence can vary from 1-3% in retrospective studies to 40% in prospective studies depending on the definition used (clinical and/or radiological). Many monocentric studies determined clinical and dosimetric risk factors associated with RILI and recommended dosimetric thresholds. Clinical risk factors reported include age >50 years, history of smoking, history of pulmonary disease, chronic infection/immune disorder, adjuvant chemotherapy, tamoxifen concomitant with RT and structural deformity of the thoracic wall (4–7). Regarding the dosimetric thresholds, there are no unanimously recognized recommendations for the ipsilateral lung in the context of breast cancer treatment (8–13). The meta-analysis of Gokula et al. (14), recommended thresholds of V20 Gy not exceeding 30% and Dmean not exceeding 15 Gy. Other studies found that locoregional lymph nodes irradiation is a strong clinical risk factor since it results in higher doses delivered to the lung adjacent to the lymph node areas (8).

The CANTO RT cohort provides a unique opportunity to assess long-term outcomes and toxicities of breast cancer RT over time (15). CANTO-RT is a subset of CANTO (NCT01993498) a prospective longitudinal multicentric cohort that enables clinical and dosimetric risk factor assessment for long term RT toxicities (16, 17).

The primary aim of our study was to assess the incidence of RILI after RT treatment in CANTO-RT and to identify clinical and dosimetric risk factors associated with RILI occurrence. The secondary aim was to compare former dosimetric thresholds proposed in the literature, and to select the most appropriate in our multicentric dataset to apply for clinical practice.

## Material and methods

This work is an etiological cross-sectional study based on the prospective CANTO-RT cohort, a subset of the CANTO cohort (NCT01993498).

CANTO Inclusion and exclusion criteria were previously published (18). Briefly, patients were women aged 18 years and over, treated for invasive cT0-3 cN0-3 M0 breast cancer, and completed 5-year follow-up for side effects, based on questionnaires and medical visits. All patients included in our study where part the CANTO-RT, which colligated full DICOM data on RT modalities and dosimetric analysis as previously described (15). Data from the patients recruited in the three larger CANTO-RT centers were included in our study.

**Figure 1:**
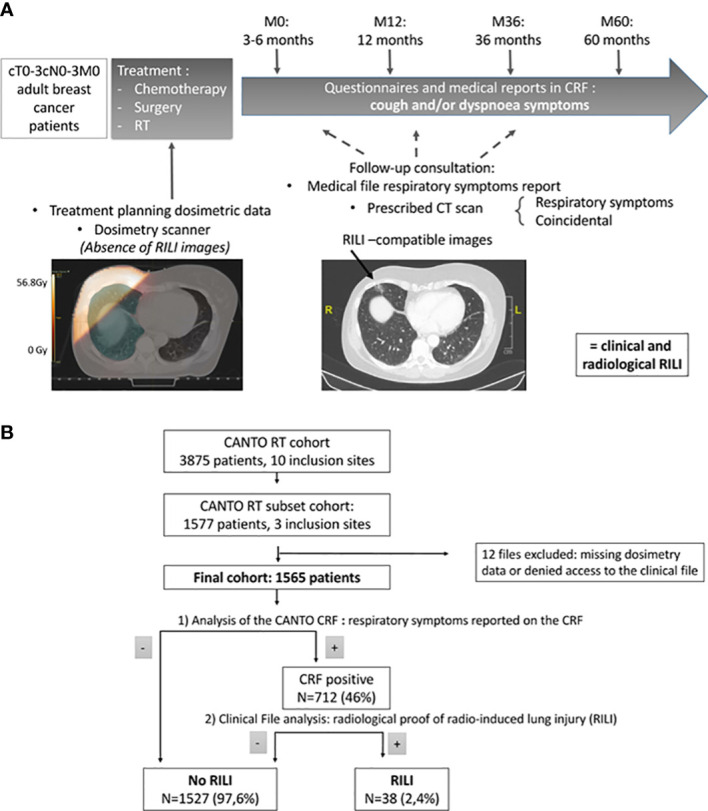
**(A)** Study design. CRF= Clinical Report Form **(B)** Flowchart of the study. Out of the 1577 patients from the three main recruiting centers of CANTO, 12 were excluded and 1565 included our study. Of these patients, 712 had dyspnea, cough, or both recorded in the CRF. After analysis of the medical files, 38 proven RILI were found, of which 17 (45%) were diagnosed by the clinicians and 21 (55%) were identified on medical imaging.

RILI identification was initially based on Case Report Form (CRF) analysis, to identify patients that presented dyspnea, cough, or both dyspnea and cough at either M0 (3 to 6 months after completion of RT), M12 (12 months after the completion of RT), M36 or M60. Events were graded according to the Radiation Therapy Oncology Group (RTOG) classification: Grade I: asymptomatic or mild symptoms, Grade II: moderate symptoms, Grade III: severe symptoms requiring oxygen therapy, Grade IV: symptoms requiring assisted ventilation, and Grade V: death. The medical files of symptomatic patients were then retrospectively analyzed, and patients were considered having RILI when a CT scan or a chest X-Ray identified compatible pulmonary lesions during the follow-up. RILI was considered acute when the symptoms were declared at M0 and chronic at later time points. An event was defined as RILI when both radiological and clinical symptoms were recorded in a medical consultation report; however, when mentioned only in the medical imaging report, often performed for other causes years after the end of treatment, RILI was not considered as diagnosed by the physician. RILI events were then retrospectively graded according to the RTOG criteria. Absence of pre-existing fibrosis, which could mislead to RILI diagnosis, was confirmed by reviewing the dosimetric CT scans (**Figure 1A**).

RT treatments were performed with LINAC accelerators, using photons with or without electrons beams. RT was mainly performed with 3D conformational RT, however intensity-modulated radiation therapy (IMRT) was allowed. Treatment plans were performed with various Treatment Planning Systems that could use type A (e.g. Clarkson) or type B (e.g. Collapsed Cone Convolution) algorithms. For patients without nodal irradiation, whole breast irradiation was delivered in dorsal or lateral decubitus (19). Dose constraints on the ipsilateral lung and field arrangement were at the discretion of the treating centers (**Supplementary Table S1**). DIBH technique was allowed to minimize the dose to the heart and lungs. Fractionation scheme were normofractionnated (NF RT) with 50 Gy in 25 fractions over 5 weeks or moderately hypofractionated (HF RT) with 40 Gy in 15 fractions over 3 weeks.

Statistical analysis was performed using R software, version 4.0.4, with the “pROC” package for ROC curve analysis and the “arsenal” package for descriptive table analyses.

RILI+ (patients with RILI) and RILI- (patients without RILI) groups were compared by the Wilcoxon test for continuous characteristics and a Chi2 or Fisher test was used to compare proportions of categorical variables. Correlations between dosimetric parameters were tested using Spearman’s correlation analysis. Statistical analysis did not include missing data. Dosimetric threshold analysis was performed by building contingency tables and calculating the positive and negative predictive values and multivariable analysis were performed using a logistic regression with backwards elimination selection. Models were compared using the Akaike information criterion (AIC) and the Bayesian information criterion (BIC). Some publications outlined that dosimetric threshold definition using ROC curves and univariable models may be less accurate than multivariable model-based threshold assessment and proposed iterative multivariable-model-based threshold definition (20). Such an analysis was thus also performed.

All patients provided a written informed consent to participate in the CANTO cohort. The study protocol was approved by a central ethical committee for human subjects (n° ID RCB: 2011-A01095-36).

## Results

### Events identification

Of the 1577 patients with data collected at three centers, 12 were excluded because of lack of dosimetric data or medical records, and 1565 were included in this study. The median follow-up time was 5.2 [range: 0.35- 8.3] years.

The initial analysis of the CANTO CRF showed 712/1565 patients (46%) with respiratory symptoms (cough, dyspnea, or both) within five years after treatment completion. Of these patients, 20% were asked to perform pulmonary imaging during their follow-up (chest X-Ray or CT scan). Among them, 38 (2.4% of total patients) presented pulmonary damage on imaging compatible with a RILI (mainly fibrous scars ipsilateral to the treatment fields, one bronchiolitis obliterans organizing pneumonia (BOOP)) (**Figure 1B**).

RILI events were mainly grade I (N=21, 55%) or grade II (N=15, 39%). Only 1 (3%) event was grade III and 1 (3%) grade IV. No grade V event was observed. Overall 2/1565 (0.1%) patients presented a grade III or higher event; none of these 2 patients had previous medical history.

The symptoms declared on the CRF were dyspnea (N=26, 68%), cough (N=4, 11%), and dyspnea and cough (N=8, 21%). Among the 38 patients identified with a RILI after their treatment, 17 (45%) were diagnosed and 21 (55%) not diagnosed by the physician. Only 8 patients (21%) required treatment for RILI. Among RILI presenting patients, RILI was acute in 13 patients (34%) and late-stage in 25 patients (66%).

### Population characteristics

**Table 1** shows patient, disease, and treatment characteristics in the overall population and according to the presence (RILI+) or absence (RILI-) of RILI.

**Table 1:** Summarized population and treatment characteristics at inclusion.

	Overall (N=1565)	RILI+ (N=38)	RILI- (N=1527)	P-values
Age, yrs				0.37 (3)
Median (Range)	55.5 (23.3, 84.4)	54.3 (27.6, 74.1)	55.5 (23.3, 84.4)	
Missing	10	0	10	
**Cardiovascular disease record, N (%)**				**0.03 (1)**
No	1070 (70%)	32 (86%)	1038 (70%)	
Yes	449 (30%)	5 (14%)	444 (30%)	
Missing	46	1	45	
**Respiratory disease record, N (%)**				**< 0.01 (1)**
No	1314 (87%)	26 (70%)	1288 (87%)	
Yes	205 (13%)	11 (30%)	194 (13%)	
Missing	46	1	45	
* Type of respiratory history :				
Asthma	105 (7%)	3 (8%)	103 (7%)	0,74 (1)
COPD	15 (1%)	2 (5%)	13 (1%)	0,053 (1)
Tuberculosis	17 (1%)	1 (3%)	16 (1%)	0,34 (1)
History of acute pneumonia	17 (1%)	2 (5%)	15 (1%)	0,07 (1)
Interstitial syndrome	3 (0,1%)	0 (0%)	3 (0%)	1 (1)
other	87 (6%)	4 (11%)	66 (4%)	0,10 (1)
Patients with multiple respiratory diseases	20 (1%)	1 (3%)	19 (1%)	0,39 (1)
Tumor Size (pT)*, N (%)				**< 0.01 (2)**
0	14 (1%)	0 (0%)	14 (1%)	
1	1042 (67%)	16 (42%)	1026 (68%)	
2	421 (27%)	17 (45%)	404 (27%)	
3	78 (5%)	5 (13%)	73 (5%)	
Missing	10	0	10	
Nodal Status (pN)*, N (%)				**< 0.01 (2)**
0	1035 (66%)	16 (42%)	1019 (67%)	
1	408 (26%)	14 (37%)	394 (26%)	
2	85 (5%)	6 (16%)	79 (5%)	
3	35 (2%)	2 (5%)	33 (2%)	
Missing	2	0	2	
**Type of Surgery, N (%)**				**0.02 (2)**
Tumorectomy	1218 (80%)	25 (66%)	1243 (79%)	
Mastectomy	298 (20%)	12 (32%)	310 (20%)	
Bilateral Mastectomy	3 (0%)	0 (0%)	3 (0%)	
Bilateral Tumorectomy	7 (0%)	0 (0%)	7 (0%)	
Tumorectomy and Mastectomy	1 (0%)	1 (3%)	2 (0%)	
**Chemotherapy, N (%)**				**< 0.01 (1)**
No	724 (46%)	9 (24%)	715 (47%)	
Yes	839 (54%)	29 (76%)	810 (53%)	
Missing	2	0	2	
**Nodal area radiation therapy, N (%)**				**< 0.01 (1)**
No	1004 (64%)	15 (39%)	989 (65%)	
Yes	561 (36%)	23 (61%)	538 (35%)	
Missing	0	0	0	
**Internal mammary chain radiation therapy, N (%)**				**< 0.01 (1)**
No	1085 (69%)	17 (45%)	1068 (70%)	
Yes	480 (31%)	21 (55%)	459 (30%)	
Missing	0	0	0	

1. Pearson’s Chi-squared test.

2. Fisher’s Exact Test for Count Data.

3 Wilcoxon test.

*TNM 7 (2010) version, COPD: Chronic Obstructive Pulmonary Disease. N: number of patients. RILI+: Radio-Induced Lung Injury presenting patients. RILI-: Radio-Induced Lung Injury negative patients. Bold values show statistically significant variables.

For the overall population, the median age was 55.5 years (range: 23.3-84.4) (**Table 1**). The median follow-up time was 5.2 years [range: 0.35-8.3].

No significant differences were found between patient characteristics, however, 11/38 RILI+ patients (30%) presented a record of respiratory disease whereas only 194/1527 RILI- patients (13%) had such a history (**Supplementary Table S2**). Respiratory history showing a trend for association with RILI occurrence were Chronic Obstructive Pulmonary Disease (COPD, p=0.05) and medical history of acute pneumonia before treatment (p= 0.07) (**Table 1**). Dosimetric CT scan showed that 3 patients presenting RILI had mild interstitial syndrome before RT. These patients presented a grade I, not diagnosed RILI, detected several years after RT. Both presented a known respiratory disease record of COPD and Sleep apnea.

Regarding tumor characteristics (**Supplementary Table S3**), the only two factors associated with RILI occurrence were a larger tumor size and nodal involvement (p<0.01).

In terms of treatment characteristics, fractionation was available for 840 patients (54% of the study population) of whom 751 patients (89%) were treated with NF RT +/- boost and 89 (11%) with HF RT (**Supplementary Table S4**). There was no difference in RILI occurrence between the three investigating centers, RT was mainly performed in conformational 3D (96% of total population) and there was no difference in RT Boost used between the RILI+ and RILI- groups. Both groups presented the same frequency of endocrine therapy and trastuzumab treatment delivered after RT. In contrast, RILI+ patients had more frequently received chemotherapy (adjuvant or neoadjuvant) (76% of RILI + versus 53% of RILI - patients (p<0.01)) (**Supplementary Table S4**). Yet, no differences were observed in terms of chemotherapy regimens (**Supplementary Table S5**). RILI + and RILI- patients had different surgery types (p=0.02); there were 25 (66%) RILI+ patients with mastectomy and 12 (32%) RILI+ patients with tumorectomy. RILI+ patients received more often RT to all nodal levels and more specifically to the internal mammary chain (p<0.01) (**Supplementary Table S4**).

### Dosimetric analysis

The dosimetric analysis showed that the doses of radiation received by the ipsilateral lung was higher for RILI+ than RILI- patients, regardless of the parameters analyzed (from V5 Gy to V40 Gy and Dmean) (**Table 2**).

**Table 2:** Dosimetric parameters of the treatment plans.

	Overall (N=1565) Median (Range)	RILI+ (N=38) Median (Range)	RILI- (N=1527) Median (Range)	P-values*
**V5 Gy (% of ipsilateral lung)**	22.2 (0.0, 100.0)	45.8 (0.0, 91.8)	21.9 (0.0, 100.0)	**< 0.01**
**V10 Gy (% of ipsilateral lung)**	15.4 (0.0, 95.6)	30.2 (0.0, 58.5)	15.2 (0.0, 95.6)	**< 0.01**
**V15 Gy (% of ipsilateral lung)**	13.0 (0.0, 70.3)	23.4 (0.0, 47.9)	12.9 (0.0, 70.3)	**< 0.01**
**V20 Gy (% of ipsilateral lung)**	11.8 (0.0, 62.1)	20.8 (0.0, 38.5)	11.6 (0.0, 62.1)	**< 0.01**
**V25 Gy (% of ipsilateral lung)**	10.8 (0.0, 56.0)	19.4 (0.0, 36.6)	10.6 (0.0, 56.0)	**< 0.01**
**V30 Gy (% of ipsilateral lung)**	9.6 (0.0, 54.3)	17.2 (0.0, 34.1)	9.4 (0.0, 54.3)	**< 0.01**
**V35 Gy (% of ipsilateral lung)**	8.0 (0.0, 51.9)	13.1 (0.0, 29.7)	7.9 (0.0, 51.9)	**< 0.01**
**V40 Gy (% of ipsilateral lung)**	5.3 (0.0, 49.7)	8.1 (0.0, 17.5)	5.2 (0.0, 49.7)	**< 0.01**
**Dmean (Gy)**	6.9 (0.0, 29.0)	11.6 (0.7, 18.8)	6.8 (0.0, 29.0)	**< 0.01**

*P-value from a Wilcoxon test. Vx Gy: % of ipsilateral lung volume receiving x Gy. Dmean: mean dose to the ipsilateral lung (Gy). RILI+: Radio-Induced Lung Injury presenting patients, RILI-: Radio-Induced Lung Injury negative patients. Presented values are numerical and not EQD2. No missing dosimetric data, Bold values show statistically significant variables.

We then assessed differentially expressed parameters between the RILI+ and RILI- populations. In a univariable analysis, the dosimetric criteria the most predictive of RILI occurrences were V30 Gy (odds ratio [OR]=1.08, 95% confident interval[CI]=1.04-1.12, p<0.001), V35 Gy (OR=1.1, 95% CI=1.05-1.15, p<0.001), V40 Gy (OR=1.09, 95% CI=1.03-1.15, p=0.002) and Dmean (OR=1.09, 95% CI=1.03-1.15, p=0.002) (**Supplementary Table S6**).

To further outline parameters that would be predictive of RILI occurrence in our dataset, we analyzed the performance of formerly published dosimetric thresholds in our population. Using contingency tables (**Supplementary Table S7**), we validated that the majority of these published (8, 9, 11–14) threshold (V20 Gy > 20% (OR=10.0, p=0.002), V25 Gy > 10% (OR=7.1, p=0.007), V30 Gy >10% (OR=12.3, p=0.0004), V30 Gy > 20% (OR=5.4, p=0.02), Dmean > 15Gy (OR=10.1, p=0.001)) correlated with RILI occurrence in our dataset (**Supplementary Table S8**). This observation was in accordance with the fact that all dosimetric parameters were highly correlated with each other (**Supplementary Table S9**).

**Figure 2:**
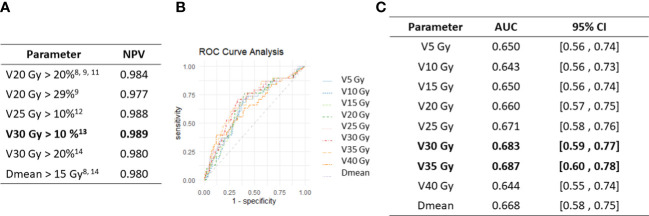
Univariable dosimetric thresholds and ROC curves analysis. **(A)** Analysis of the negative predictive values (NPV) of dosimetric thresholds previously published (8, 9, 11–14). **(B)** ROC curves of the different dosimetric parameters studied. **(C)** ROC curve AUC measured for all dosimetric parameters in our data set.

We then analyzed the negative predictive value (NPV) associated with those different dosimetric thresholds (i.e. the probability of not developing a RILI when the treatment plan respects the threshold). Interestingly, the threshold of V30 Gy > 10% had the highest NPV in our dataset (NPV=0.989) (**Figure 2A**).

ROC curves showed that V30 Gy and V35 Gy had the highest AUC and were thus the more predictive of RILI occurrence in our dataset (**Figures 2B, C**).

### Adjusted risk of clinical and dosimetric factors

We then analyzed RILI occurrence regarding dosimetric parameters and the main clinical characteristics of patients (respiratory disease record, nodal area involvement and associated chemotherapy) in a multivariable analysis using the AIC and BIC indicators to compare the different multivariable models. V30 Gy (OR=1.06, 95% CI=1.01-1.12, p=0.04) and respiratory disease record (OR=3.05, 95% CI= 1.47-6.34, p<0.01) or V35 Gy (OR=1.07, 95% CI =1.01-1.14, p=0.02) and respiratory disease record (OR=3.07, 95% CI=1.48-6.38, p<0.01) remained significantly associated with RILI occurrence in the multivariable analysis adjusted for nodal area involvement and associated chemotherapy, showing the lowest AIC and BIC values (**Table 3**).

**Table 3:** Multivariable analyses of RILI – associated factors (Logistic Regression).

Variables	n/N	OR	95% CI	P values	AIC	BIC
**Baseline respiratory disease** Chemotherapy Nodal RT Dmean	**11/205** 29/839 23/561	**3.04**1.67 1.96 1.06	**[1.46, 6.31]** [0.70, 4.01] [0.89, 4.34] [0.99, 1.13]	**<0.01** 0.25 0.10 0.08	335	361
**Baseline respiratory disease** Chemotherapy Nodal RT V 5 Gy	**11/205** 29/839 23/561	**2.97** 1.80 1.70 1.01	**[1.43,6.16]** [0.75, 4.31]v[0.52, 5.54] [0.99, 1.03]	**<0.01** 0.19 0.38 0.49	337	364
**Baseline respiratory disease** Chemotherapy Nodal RT V 10 Gy	**11/205** 29/839 23/561	**2.95** 1.84 2.20 1.01	**[1.42,6.13]** [0.77, 4.41] [0.72, 6.75] [0.98, 1.03]	**<0.01** 0.17 0.17 0.90	338	364
**Baseline respiratory disease** Chemotherapy Nodal RT V 15 Gy	**11/205** 29/839 23/561	**3.00** 1.81 1.78 1.01	**[1.45,6.24]** [0.76, 4.33] [0.57, 5.58] [0.97, 1.05]	**<0.01** 0.18 0.32 0.53	337	364
**Baseline respiratory disease** Chemotherapy Nodal RT V 20 Gy	**11/205** 29/839 23/561	**3.05** 1.77 1.43 1.03	**[1.47, 6.34]** [0.74, 4.23] [0.46, 4.45] [0.98, 1.08]	**<0.01** 0.20 0.54 0.24	336	363
**Baseline respiratory disease** Chemotherapy Nodal RT V 25 Gy	**11/205** 29/839 23/561	**3.07** 1.74 1.25 1.04	**[1.48, 6.38]** [0.73, 4.16] [0.42, 3.72] [0.99, 1.10]	**<0.01** 0.22 0.69 0.11	335	361
**Baseline respiratory disease** Chemotherapy Nodal RT V 30 Gy	**11/205** 29/839 23/561	**3.05** 1.72 1.21 1.06	**[1.47, 6.34]** [0.72, 4.10] [0.44, 3.30] [1.01, 1.12]	**<0.01** 0.23 0.71 0.04	334	360
**Baseline respiratory disease** Chemotherapy Nodal RT V 35 Gy	**11/205** 29/839 23/561	**3.07** 1.68 1.38 1.07	**[1.48, 6.38]** [0.70, 4.01] [0.57, 3.38] [1.01, 1.14]	**<0.01** 0.25 0.48 0.02	333	360
**Baseline respiratory disease** Chemotherapy Nodal RT V 40 Gy	**11/205** 29/839 23/561	**3.04** 1.67 1.96 1.06	**[1.46, 6.31]** [0.70, 4.01] [0.89, 4.34] [0.99, 1.13]	**<0.01** 0.25 0.10 0.08	335	361

n/N, number of RILI/number of patients; OR, Odds Ratio CI: Confidence Interval; RT, Radiation therapy; AIC, Akaike information criterion; BIC, Bayesian information criterion. Vx Gy: % of ipsilateral lung volume receiving x Gy. Dmean: mean dose to the ipsilateral lung (Gy); * 46 patients excluded for missing data for baseline respiratory disease (n=46) and for chemotherapy (n=2). Presented values are numerical and not EQD2. Bold values show statistically significant variables.

We then performed iterative multivariable model threshold assessment for V30 Gy and V35 Gy parameters (**Table 4**). V30 Gy > 15% (OR=3.07, CI=1.04-9.05, p=0.03) and V35 Gy > 25% (OR=6.45, CI=1.25-33.17, p=0.03) were significantly associated with RILI occurrence in a multivariable model adjusted for baseline respiratory disease, chemotherapy and nodal RT, V30 Gy > 15% of ipsilateral lung had the lowest AIC and BIC values (AIC=333, BIC=360) (**Table 4**). When performing the same analysis with all the dosimetric thresholds, Dmean > 10 Gy, V20 Gy > 15%, V25 Gy > 15%, V40 Gy > 10% were also significantly associated with RILI occurrence (**Supplementary Table S10**).

**Table 4:** Multivariable analyses of V 30 Gy and V 35 Gy dosimetric thresholds (Logistic Regression).

Variables	n/N	OR	95% CI	P values	AIC	BIC
**Baseline respiratory disease** Chemotherapy Nodal RT V30 Gy > 5%	**11/205** 29/839 23/561 34/1141	**2.99**1.76 1.93 1.91	**[1.44, 6.19]** [0.74, 4.19] [0.85, 4.35] [0.60, 6.05]	**<0.01** 0.20 0.12 0.27	336	363
**Baseline respiratory disease** Chemotherapy Nodal RT V30 Gy > 10%	**11/205** 29/839 23/561 29/734	**3.09** 1.72 1.34 2.59	**[1.49, 6.42]** [0.72, 4.12] [0.52, 3.32] [0.99, 6.75]	**<0.01** 0.22 0.52 0.05	334	361
**Baseline respiratory disease** Chemotherapy Nodal RT V30 Gy > 15%	**11/205** 29/839 23/561 23/457	**3.05** 1.77 1.01 3.07	**[1.47,6.35]** [0.74, 4.24] [0.32, 3.15] [1.04, 9.05]	**<0.01** 0.19 0.98 0.03	333	360
**Baseline respiratory disease** Chemotherapy Nodal RT V30 Gy > 20%	**11/205** 29/839 23/561 10/198	**2.92** 1.83 2.02 1.47	**[1.41,6.05]** [0.77, 4.39] [0.87, 4.7] [0.63, 3.42]	**<0.01** 0.17 0.10 0.37	337	364
**Baseline respiratory disease** Chemotherapy Nodal RT V30 Gy > 25%	**11/205** 29/839 23/561 3/45	**2.98** 1.84 2.19 1.93	**[1.43, 6.17]** [0.77, 4.40] [0.99, 4.81] [0.54, 6.87]	**<0.01** 0.17 0.05 0.31	337	364
**Baseline respiratory disease** Chemotherapy Nodal RT V35 Gy > 5%	**11/205** 29/839 23/561 33/1044	**3.04** 1.71 1.84 2.11	**[1.46, 6.30]** [0.72, 4.07] [0.82, 4.13] [0.74, 6.05]	**<0.01** 0.23 0.14 0.16	336	362
**Baseline respiratory disease** Chemotherapy Nodal RT V35 Gy > 10%	**11/205** 29/839 23/561 25/585	**3.02** 1.76 1.52 2.15	**[1.45, 6.27]** [0.73, 4.20] [0.62, 3.72] [0.92, 5.01]	**<0.01** 0.21 0.36 0.08	335	361
**Baseline respiratory disease** Chemotherapy Nodal RT V35 Gy > 15%	**11/205** 29/839 23/561 14/239	**2.92** 1.81 1.77 1.94	**[1.41, 6.07]** [0.75, 4.32] [0.75, 4.20] [0.87, 4.34]	**<0.01** 0.18 0.19 0.11	335	362
**Baseline respiratory disease** Chemotherapy Nodal RT V35 Gy > 20%	**11/205** 29/839 23/561 2/40	**3.04** 1.67 1.96 1.37	**[1.46, 6.31]** [0.70, 4.01] [0.89, 4.34] [0.31, 6.15]	**<0.01** 0.25 0.10 0.68	338	364
**Baseline respiratory disease** Chemotherapy Nodal RT V35 Gy > 25%	**11/205** 29/839 23/561 2/11	**3.00** 1.84 2.16 6.45	**[1.44, 6.23]** [0.77, 4.4] [0.99, 4.74] [1.25, 33.17]	**<0.01** 0.17 0.05 0.03	334	361

n/N, number of RILI/number of patients; OR, Odds Ratio CI, Confidence Interval; RT, Radiation therapy; AIC, Akaike information criterion; BIC, Bayesian information criterion. Vx Gy: % of ipsilateral lung volume receiving x Gy. Dmean: mean dose to the ipsilateral lung (Gy); * 46 patients excluded for missing data for baseline respiratory disease (n=46) and for chemotherapy (n=2). Presented values are numerical and not EQD2. Bold values show statistically significant variables.

## Discussion

Our analysis on 1565 patients within the multicentric prospective longitudinal CANTO-RT cohort estimated a RILI incidence of 2.4% after early breast cancer treatment, with only half of the events prospectively identified by the physicians. The respiratory adverse events were mainly grade I-II (95%) with no grade V, confirming treatment safety regarding RILI. The main clinical predictor was respiratory disease history, especially COPD or medical history of acute pneumonia before treatment. All dosimectric parameters analyzed were higher in RILI+ patients and highly correlated to each other. ROC curves analysis showed a strong prediction for all Vx in ipsilateral lung with V30 Gy and V35 Gy being the most robust factors. Those factors and respiratory disease history remained significantly associated with RILI occurrence in multivariable analysis adjusted for chemotherapy and nodal RT. We found V30 Gy > 15% was strongly associated with RILI occurrence in CANTO RT dataset.

We noted close predictive values for all the dosimetric parameters analysed. This is in accordance with the formerly published papers showing different dosimetric parameters as the most predictive of RILI occurrence. However, our results substantiate data from the monocentric study conducted in an Asian population by Lee et al. (13). These results are important since the Asian population can have different pulmonary responses to exogenous agents compared to the Caucasian population (21). Concerning the V30 Gy parameter, V30 Gy > 10%, published by Lee et al. (13) is close to our threshold of V30 Gy >15%. In the study of Lee et al. (13), RT was performed with IMRT in 44% of cases, mainly with a moderately hypofractionnated regimen with doses reported in EQD2. In our series, IMRT use was limited and hypofractionnation could not be analysed due to missing data.

Other studies recommended V20 Gy as the best predictor for RILI occurrence. Few studies compared V20 Gy and V30 Gy concluding in favor of V20 Gy (9, 22), some analyzed V20 Gy and V30 Gy independently (8). Other studies recommended the V20 Gy parameter in comparison to V10 Gy (4) or V5 Gy (23), but not to V30 Gy. In a study performing prospective pulmonary function test and CT scans, V20 Gy was more predictive than V30 Gy for RILI occurrence in a multivariable analysis with an AUC of 0.69 for V20 Gy, when it was 0.67 for V30 Gy and 0.68 for V30 Gy in our study (22). One study dichotomized dosimetric results between associated and non-associated nodal RT; V20 Gy not exceeding 38% was optimal with nodal RT and V20 Gy not exceeding 20% without nodal RT. This study analyzed also V30 Gy in the subgroup with nodal RT only and showed a slightly higher AUC and a threshold of V30 Gy not exceeding 25.7% compared to V20 Gy (11).

Our backwards multivariable analysis showed that regional nodes irradiation, including IMC, tumor size, nodal status, chemotherapy use and the type of surgery association to RILI were eventually all related to the dose delivered to the lung, as among them the dose is the only significant factor in the multivariable model. Clinical parameters such as age and smoking status (22, 23) were not predictive of RILI appearance in our dataset. In addition, tamoxifen use after RT was not predictive of RILI appearance, showing the safety of its use after RT completion. No concomitant tamoxifen use was noted in the cohort. We did not find any association between the type of chemotherapy regimen used and RILI, in accordance with former studies that have not find any difference in respiratory function decline after breast chemotherapy followed by RT, regardless of chemotherapy drug types (24). Cardiovascular history was less frequent in RILI+ than RILI- patients, which might be explained by the use of more stringent dose constraints to the thorax in such patients, specific volume delineation or specific technique (such as lateral decubitus).

We believe the methodology used for event detection was reliable due to the extensive and accurate two-step cross analysis based on the CRF and the individual medical record verification of the symptomatic events. All the events had imaging proof of new lung injury after RT treatment, limiting the risk of false positive. The incidence of RILI obtained by our two-step analysis is compatible with those found in retrospective studies (25–28).

Four patients presented RILI with surprisingly low doses to the ipsilateral lung. However, RILI occurrence was proven for at least one of them. Indeed, this patient underwent multiple chest CT scans, demonstrating the absence of radiographic event shortly after treatment completion. The patient had a medical history of previous chemotherapy and mediastinal RT for a Hodgkin lymphoma. It is therefore plausible that, for this patient, the low doses of RT received by the lung during the breast cancer treatment, plus the previous doses received during the treatment of Hodgkin lymphoma, resulted in a cumulative dose high enough to induce RILI. We cannot exclude that other patients with low doses to the ipsilateral lung presented unknown risk factors for RILI, suggesting that dosimetric parameters might incompletely predict the risk of RILI.

The main pitfalls of our study are the retrospective analysis of medical records to identify RILI events, although the use of the prospective CANTO CRF allowed us to identify twice as many events as we would have found with medical file analysis alone. Indeed, only half of the events were diagnosed by the physician and therefore reported as such in the clinical file. RILI assessment with systematic imaging usually results in higher proportion of events, the majority of which, however, are asymptomatic with up to 58% grade I and 16% grade II (14, 25, 29).

As RILI detection was not initially planned in CANTO, no systematic imaging was done. We added a retrospective data collection from patient clinical files and imaging from three of the top recruiting centers of CANTO-RT. We thus acknowledge several possible biases introduced by the definition of RILI events that we had to set.

First, our definition of RILI was based on cough and dyspnea presence on the CRF. Those symptoms are pathognomonic of RILI but not diagnosis-defining. We could have missed RILI events with a clinical presentation of fatigue, low-grade fever, chest pain without cough and dyspnea (2). Then, in some patients with underlying pulmonary disease, a modest increase in symptom severity might be present without it being classified as new cough or dyspnea. Hence, it can be suspected that a minority of patients presenting RILI (most likely not clinically meaningful) were not recognized by this method. To better capture these events, monitoring of patient reported outcome measures (PROMs) could have been used.

There was no systematic chest CT among the study population, which might introduce a confounding bias. Patients with underlying pulmonary condition could have been more prone to undergo a CT scan during the follow-up and more RILI could have been detected in those patients. The reason for radiological imaging in those patients was not always recorded but when mentioned in the medical files, it was usually the occurrence of cough, dyspnea or chest pain, or the suspicion of a metastatic evolution.

As only 20% of patients with reported respiratory symptoms on the CRF performed a pulmonary imaging, we may have underestimated the true incidence of RILI grade I events in our population. We also acknowledge that our study did not perform respiratory function tests to identify infra-radiological events, a method that could possibly results in the detection of additional RILI cases (30, 31). In addition, our study design does not enable to analyze RILI occurring more than 5 years after treatment.

Data on fractionation schedule were only available in 54% of the study population, and there was an incomplete record in some centers, resulting in an uneven distribution of the missing data among the three centers. Consequently, we could not analyse the dosimetric data taking into account the fractionation scheme. This was mostly due to the lack of contouring guidelines in CANTO, resulting in the absence of contouring of the target volume in some centers at the time of CANTO beginning. Our study remained possible since lung contours were lacking in only 12 patients out of 1577 in the three centers studied (as shown in **Figure 1A**).

To try to assess the impact of fractionation information absence, taking into account the 3 treating centers local guidelines for the indication of hypofractionated radiation therapy, we approximated the fractionation schedule received by the patients based on age and nodal status. Patients were considered having an hypofractionated regimen (40 Gy in 15 fractions) when under 50 years old and having no nodal involvement leading to a total number of 825 patients treated with normofractionated radiation therapy and 733 treated with hypofractionated radiation therapy. Assuming an alpha/beta around 3 we considered the EQD2 (dose equivalent to a 2 Gy by fraction scheme) for lung depicted in **Supplementary Table S11** (32). In this analysis, V30 stayed a better dosimetric predictor of RILI than V5 Gy or V20 Gy on the ROC curve analysis (**Supplementary Table S11**) and it was still associated with RILI occurrence in multivariable analysis (**Supplementary Table S12**).

RILI events were rare in respect to the general population (38/1565), which limited statistical analysis. Most events were low grade but subgroup analysis of grade III-IV RILI (n=2) or RILI requiring treatment (n=8) was not performed due to small sample size.

We could not directly compare 3D RT to IMRT or VMAT since treatment was performed by conformational 3D RT for 96% of patients. In rotational techniques, the larger lung volumes receiving low-doses may imply different dosimetric thresholds for RILI occurrence (33, 34). Type A or type B calculation algorithms could be used for treatment planning. Type B algorithms are known for higher dose heterogeneity and more precise dose assessment, with statistically larger high-dose volumes (35). In our studies, we cannot evaluate the impact of the Treatment Planning Systems or algorithm types used on the dosimetric results as they were not recorded.

In conclusion, this study is to our knowledge one of the largest multicentric study to analyze the dosimetric parameters associated with RILI occurrence after early breast cancer treatment including 3D RT. Mild respiratory symptoms were frequently reported by CANTO patients. Lung medical imaging was not systematic. Radiologically confirmed RILI were observed in 2.4% of patients (n=38). To further improve lung protection during 3D conformational breast RT, we recommend V30 Gy not exceeding 15% threshold, especially in patients presenting a medical history of respiratory disease such as COPD or acute pneumonia before treatment.

Further studies are needed to provide multicentric data with IMRT including tomotherapy and VMAT and hypofractionated regimens to assess dosimetric risk factors for RILI with modern breast RT.

## Data availability statement

The data analyzed in this study is subject to the following licenses/restrictions: CANTO-RT dataset published in Sarrade and al, Cancers 2023. Requests to access these datasets should be directed to sofia.rivera@gustaveroussy.fr.

## Author contributions

AG: Data collection, statistical analysis, manuscript redaction TS: CANTO-RT database completion. YK, BD, FD, GA, AM, SE, NM, CB, AB, TL, DP, SeR, AM, FP, FA, ED: CANTO data collection and manuscript editing. BD: Advise regarding clinical RILI description. RA: Statistical analysis and manuscript redaction supervision. SR: Study design and manuscript redaction supervision. All authors contributed to the article and approved the submitted version.

## References

[1] Deng G Liang N Xie J Luo H Qiao L Zhang J. Pulmonary toxicity generated from radiotherapeutic treatment of thoracic malignancies. Oncol Lett (2017) 14(1):501−11. doi: 10.3892/ol.2017.6268 28693198 PMC5494764

[2] Arroyo-Hernández M Maldonado F Lozano-Ruiz F Muñoz-Montaño W Nuñez-Baez M Arrieta O. Radiation-induced lung injury: current evidence. BMC Pulm Med (2021) 21(1):9. doi: 10.1186/s12890-020-01376-4 33407290 PMC7788688

[3] Marks LB Bentzen SM Deasy JO Kong FM Bradley JD Vogelius IS. Radiation dose–volume effects in the lung. Int J Radiat Oncol Biol Phys (2010) 76(3):S70−6. doi: 10.1016/j.ijrobp.2009.06.091 20171521 PMC3576042

[4] Kubo A Osaki K Kawanaka T Furutani S Ikushima H Nishitani H. Risk factors for radiation pneumonitis caused by whole breast irradiation following breast-conserving surgery. J Med Invest (2009) 56(3,4):99−110. doi: 10.2152/jmi.56.99 19763021

[5] Bentzen SM Skoczylas JZ Overgaard M Overgaard J. Radiotherapy-related lung fibrosis enhanced by tamoxifen. JNCI: J Natl Cancer Institute (1996) 88(13):918−22. doi: 10.1093/jnci/88.13.918 8656444

[6] Koc M Polat P Suma S. Effects of tamoxifen on pulmonary fibrosis after cobalt-60 radiotherapy in breast cancer patients. Radiother Oncol (2002) 64(2):171−5. doi: 10.1016/S0167-8140(02)00136-6 12242127

[7] Hanania AN Mainwaring W Ghebre YT Hanania NA Ludwig M. Radiation-induced lung injury. Chest (2019) 156(1):150−62. doi: 10.1016/j.chest.2019.03.033 30998908 PMC8097634

[8] Lind PARM Wennberg B Gagliardi G Fornander T. Pulmonary complications following different radiotherapy techniques for breast cancer, and the association to irradiated lung volume and dose. Breast Cancer Res Treat (2001) 68(3):199−210. doi: 10.1023/A:1012292019599 11727957

[9] Zhou ZR Han Q Liang SX He XD Cao NY Zi YJ. Dosimetric factors and Lyman normal-tissue complication modelling analysis for predicting radiation-induced lung injury in postoperative breast cancer radiotherapy: a prospective study. Oncotarget (2016) 8(20):33855−63. doi: 10.18632/oncotarget.12979 PMC5464917 27806340

[10] Nissen HD Appelt AL. Improved heart, lung and target dose with deep inspiration breath hold in a large clinical series of breast cancer patients. Radiother Oncol (2013) 106(1):28−32. doi: 10.1016/j.radonc.2012.10.016 23199652

[11] Wen G Tan YT Lan XW He ZC Huang JH Shi JT. New clinical features and dosimetric predictor identification for symptomatic radiation pneumonitis after tangential irradiation in breast cancer patients. J Cancer (2017) 8(18):3795−802. doi: 10.7150/jca.21158 29151967 PMC5688933

[12] Krengli M Sacco M Loi G Masini L Ferrante D Gambaro G. Pulmonary changes after radiotherapy for conservative treatment of breast cancer: a prospective study. Int J Radiat OncologyBiologyPhys (2008) 70(5):1460−7. doi: 10.1016/j.ijrobp.2007.08.050 17931797

[13] Lee BM Chang JS Kim SY Keum KC Suh CO Kim YB. Hypofractionated radiotherapy dose scheme and application of new techniques are associated to a lower incidence of radiation pneumonitis in breast cancer patients. Front Oncol (2020) 10:124. doi: 10.3389/fonc.2020.00124 32117771 PMC7026386

[14] Gokula K Earnest A Wong LC. Meta-analysis of incidence of early lung toxicity in 3-dimensional conformal irradiation of breast carcinomas. Radiat Oncol (2013) 8(1):268. doi: 10.1186/1748-717X-8-268 24229418 PMC3842634

[15] Sarrade T Allodji R Ghannam Y Auzac G Everhard S Kirova Y. CANTO-RT: one of the largest prospective multicenter cohort of early breast cancer patients treated with radiotherapy including full DICOM RT data. Cancers (2023) 15(3):751. doi: 10.3390/cancers15030751 36765709 PMC9913384

[16] Allali S Carton M Sarrade T Querel O Jacquet A Rivera S. CANTO-RT: skin toxicities evaluation of a multicentre large prospective cohort of irradiated patients for early-stage breast cancer. Int J Cancer (2022) 151(7):1098−108. doi: 10.1002/ijc.34057 35489021

[17] Ghannam Y Di Meglio A Sarrade T Jacquet A Everhard S Kirova Y. Impact of radiation therapy on fatigue at 1 year in breast cancer survivors in the prospective multicentre CANcer TOxicity cohort. Eur J Cancer (2022) 177:143−53. doi: 10.1016/j.ejca.2022.09.026 36356418

[18] Vaz-Luis I Cottu P Mesleard C Martin AL Dumas A Dauchy S. UNICANCER: French prospective cohort study of treatment-related chronic toxicity in women with localised breast cancer (CANTO). ESMO Open (2019) 4(5):e000562. doi: 10.1136/esmoopen-2019-000562 31555487 PMC6735667

[19] Kirova YM Hijal T Campana F Fournier-Bidoz N Stilhart A Dendale R. Whole breast radiotherapy in the lateral decubitus position: a dosimetric and clinical solution to decrease the doses to the organs at risk (OAR). Radiother Oncol (2014) 110(3):477−81. doi: 10.1016/j.radonc.2013.10.038 24342456

[20] El Naqa I Bradley J Blanco AI Lindsay PE Vicic M Hope A. Multivariable modeling of radiotherapy outcomes, including dose–volume and clinical factors. Int J Radiat OncologyBiologyPhys (2006) 64(4):1275−86. doi: 10.1016/j.ijrobp.2005.11.022 16504765

[21] Caldeira D Alarcão J Vaz-Carneiro A Costa J. Risk of pneumonia associated with use of angiotensin converting enzyme inhibitors and angiotensin receptor blockers: systematic review and meta-analysis. BMJ (2012) 345:e4260. doi: 10.1136/bmj.e4260 22786934 PMC3394697

[22] Lind PA Wennberg B Gagliardi G Rosfors S Blom-Goldman U Lideståhl A. ROC curves and evaluation of radiation-induced pulmonary toxicity in breast cancer. Int J Radiat OncologyBiologyPhys (2006) 64(3):765−70. doi: 10.1016/j.ijrobp.2005.08.011 16257129

[23] Palma DA Senan S Tsujino K Barriger RB Rengan R Moreno M. Predicting radiation pneumonitis after chemoradiation therapy for lung cancer: an international individual patient data meta-analysis. Int J Radiat Oncol Biol Phys (2013) 85(2):444−50. doi: 10.1016/j.ijrobp.2012.04.043 22682812 PMC3448004

[24] Saha A Chattopadhyay S. Assessment of pulmonary toxicities in breast cancer patients undergoing treatment with anthracycline and taxane based chemotherapy and radiotherapy- a prospective study. Int J Cancer Ther Oncol (2013) 1(2). doi: 10.14319/ijcto.0102.1

[25] Chitapanarux I Klunklin P Pinitpatcharalert A Sripan P Tharavichitkul E Nobnop W. Conventional versus hypofractionated postmastectomy radiotherapy: a report on long-term outcomes and late toxicity. Radiat Oncol (2019) 14(1):175. doi: 10.1186/s13014-019-1378-x 31610801 PMC6790998

[26] Matzinger O Heimsoth I Poortmans P Collette L Struikmans H Bogaert WVD. Toxicity at three years with and without irradiation of the internal mammary and medial supraclavicular lymph node chain in stage I to III breast cancer (EORTC trial 22922/10925). Acta Oncol (2010) 49(1):24−34. doi: 10.3109/02841860903352959 20100142

[27] Haviland JS Owen JR Dewar JA Agrawal RK Barrett J Barrett-Lee PJ. The UK standardisation of breast radiotherapy (START) trials of radiotherapy hypofractionation for treatment of early breast cancer: 10-year follow-up results of two randomised controlled trials. Lancet Oncol (2013) 14(11):1086−94. doi: 10.1016/S1470-2045(13)70386-3 24055415

[28] Killander F Anderson H Kjellén E Malmström P. Increased cardio and cerebrovascular mortality in breast cancer patients treated with postmastectomy radiotherapy – 25 year follow-up of a randomised trial from the south Sweden breast cancer group. Eur J Cancer (2014) 50(13):2201−10. doi: 10.1016/j.ejca.2014.04.033 24951164

[29] Tovanabutra C Katanyoo K Uber P Chomprasert K Sookauychai S. Comparison of treatment outcome between hypofractionated radiotherapy and conventional radiotherapy in postmastectomy breast cancer. Asian Pac J Cancer Prev (2020) 21(1):119−25. doi: 10.31557/APJCP.2020.21.1.119 31983173 PMC7294031

[30] Smith LM Mendenhall NP Cicale MJ Block ER Carter RL Million RR. Results of a prospective study evaluating the effects of mantle irradiation on pulmonary function. Int J Radiat OncologyBiologyPhys (1989) 16(1):79−84. doi: 10.1016/0360-3016(89)90013-8 2912958

[31] Boersma LJ Damen EMF de Boer RW Muller SH Olmos RAV van Zandwijk N. Estimation of overall pulmonary function after irradiation using dose-effect relations for local functional injury. Radiother Oncol (1995) 36(1):15−23. doi: 10.1016/0167-8140(95)01580-A 8525021

[32] Koulis TA Phan T Olivotto IA. Hypofractionated whole breast radiotherapy: current perspectives. Breast Cancer (2015) 7:363−70. doi: 10.2147/BCTT.S81710 26604820 PMC4629948

[33] Goddu SM Chaudhari S Mamalui-Hunter M Pechenaya OL Pratt D Mutic S. Helical tomotherapy planning for left-sided breast cancer patients with positive lymph nodes: comparison to conventional multiport breast technique. Int J Radiat OncologyBiologyPhys (2009) 73(4):1243−51. doi: 10.1016/j.ijrobp.2008.11.004 19251096

[34] Van Parijs H Vinh-Hung V Fontaine C Storme G Verschraegen C Nguyen DM. Cardiopulmonary-related patient-reported outcomes in a randomized clinical trial of radiation therapy for breast cancer. BMC Cancer (2021) 21(1):1177. doi: 10.1186/s12885-021-08916-z 34736429 PMC8569957

[35] Cilla S Digesù C Macchia G Deodato F Sallustio G Piermattei A. Clinical implications of different calculation algorithms in breast radiotherapy: a comparison between pencil beam and collapsed cone convolution. Physica Med (2014) 30(4):473−81. doi: 10.1016/j.ejmp.2014.01.002 24491400

