# Supplementary material for: Radiation-induced lung injury after breast cancer treatment: incidence in the CANTO-RT cohort and associated clinical and dosimetric risk factors
Source: Front Oncol. 2023 Jun 29;13:1199043. doi: 10.3389/fonc.2023.1199043 (PMC10342531; doi:10.3389/fonc.2023.1199043)
Supplement: Supplementary file 1 [file Table_1.docx]

**Table S1: Dosimetric constraints used for treatment planning in the three participating centers**

|  | **Hospital N°1** | **Hospital N°2** | **Hospital N°3** |
| --- | --- | --- | --- |
| **V30 Gy** | <20% | <10 to 18% | <15% |
| **V20 Gy** | <10% | <15 to 25% | <20% |
| **Dmean** | <18Gy | <8 to 15Gy | <10 Gy |
| **comment** |  | depending on nodal levels and CMI irradiation. | when nodal levels are irradiated, with adaptation of the doses regarding to nodal levels, boost and laterality. |

VxGy: % of ipsilateral lung volume receiving more than x Gy, Dmean: ipsilateral lung mean dose
