# Supplementary material for: Radiation-induced lung injury after breast cancer treatment: incidence in the CANTO-RT cohort and associated clinical and dosimetric risk factors
Source: Front Oncol. 2023 Jun 29;13:1199043. doi: 10.3389/fonc.2023.1199043 (PMC10342531; doi:10.3389/fonc.2023.1199043)
Supplement: Supplementary file 2 [file Table_2.docx]

**Table S2: Detailed population characteristics at inclusion**

|  | Overall (N=1565) | RILI +(N=38) | RILI - (N=1527) | p value |
| --- | --- | --- | --- | --- |
| OMS score at inclusion; N (%) |  |  |  | 1.00 (2) |
| 0 | 1287 (100%) | 33 (100%) | 1254 (100%) |  |
| 1 | 2 (0%) | 0 (0%) | 2 (0%) |  |
| Missing | 276 | 5 | 271 |  |
| Age, years |  |  |  | 0.37 (3) |
| Median (Range) | 55.5 (23.3, 84.4) | 54.3 (27.6, 74.1) | 55.5 (23.3, 84.4) |  |
| Missing | 10 | 0 | 10 |  |
| Age (By 10years increase); N (%) |  |  |  | 0.45 (2) |
| Age≤40 | 131 (8%) | 7 (18%) | 124 (8%) |  |
| 40<Age≤50 | 394 (25%) | 8 (21%) | 386 (25%) |  |
| 50<Age≤60 | 452 (29%) | 11 (29%) | 441 (29%) |  |
| 60<Age≤70 | 407 (26%) | 9 (24%) | 398 (26%) |  |
| 70<Age≤80 | 159 (10%) | 3 (8%) | 156 (10%) |  |
| 80<Age | 12 (1%) | 0 (0%) | 12 (1%) |  |
| Missing | 10 | 0 | 10 |  |
| Body Mass Index at inclusion |  |  |  | 0.87 (3) |
| Median (Range) | 24.1 (15.9, 55.1) | 24.5 (16.6, 39.0) | 24.0 (15.9, 55.1) |  |
| Missing | 14 | 0 | 14 |  |
| Smoking Status; N (%) |  |  |  | 0.19 (1) |
| Current smoker | 272 (18%) | 7 (18%) | 265 (18%) |  |
| Former smoker | 985 (64%) | 20 (53%) | 965 (64%) |  |
| Non smoker | 278 (18%) | 11 (29%) | 267 (18%) |  |
| Missing | 30 | 0 | 30 |  |
| Haemoglobin (g/dL) at inclusion |  |  |  | 0.36 (3) |
| Median (Range) | 13.6 (3.5, 39.6) | 13.7 (11.3, 15.1) | 13.6 (3.5, 39.6) |  |
| Missing | 52 | 2 | 50 |  |
| Platelets (G/L) at inclusion |  |  |  | 0.65 (3) |
| Median (Range) | 255.0 (33.8, 621.0) | 243.5 (168.0, 388.0) | 255.5 (33.8, 621.0) |  |
| Missing | 55 | 2 | 53 |  |
| Neutrophils (G/L) at inclusion |  |  |  | 0.72 (3) |
| Median (Range) | 3.9 (0.3, 144.0) | 4.3 (1.9, 9.7) | 3.9 (0.3, 144.0) |  |
| Missing | 53 | 2 | 51 |  |
| Lymphocytes (G/L) at inclusion |  |  |  | 0.70 (3) |
| Median (Range) | 1.9 (0.4, 24.5) | 1.7 (0.8, 3.7) | 1.9 (0.4, 24.5) |  |
| Missing | 54 | 2 | 52 |  |
| Creatinine Clearance at inclusion |  |  |  | 0.62 (3) |
| Median (Range) | 91.7 (35.8, 266.0) | 95.8 (45.3, 154.0) | 91.6 (35.8, 266.0) |  |
| Missing | 470 | 12 | 458 |  |
| **Cardiovascular disease record; N (%)** |  |  |  | **0.03 (1)** |
| No | 1070 (70%) | 32 (86%) | 1038 (70%) |  |
| Yes | 449 (30%) | 5 (14%) | 444 (30%) |  |
| Missing | 46 | 1 | 45 |  |
| Cancer disease record; N (%) |  |  |  | 0.96 (1) |
| No | 1435 (92%) | 35 (92%) | 1400 (92%) |  |
| Yes | 119 (8%) | 3 (8%) | 116 (8%) |  |
| Missing | 11 | 0 | 11 |  |
| Influenza vaccine record; N (%) |  |  |  | 0.78 1) |
| No | 1228 (83%) | 31 (82%) | 1197 (83%) |  |
| Yes | 247 (17%) | 7 (18%) | 240 (17%) |  |
| Missing | 90 | 0 | 90 |  |
| **Respiratory Disease Record; N (%)** |  |  |  | **< 0.01 (1)** |
| No | 1314 (87%) | 26 (70%) | 1288 (87%) |  |
| Yes | 205 (13%) | 11 (30%) | 194 (13%) |  |
| Missing | 46 | 1 | 45 |  |
| Type of respiratory history; N (%) |  |  |  |  |
| Asthma | 105 (7%) | 3 (8%) | 103 (7%) | 0,74 (1) |
| COPD | 15 (1%) | 2 (5%) | 13 (1%) | 0,053 (1) |
| Tuberculosis | 17 (1%) | 1 (3%) | 16 (1%) | 0,34 (1) |
| Pneumonia | 17 (1%) | 2 (5%) | 15 (1%) | 0,07 (1) |
| Interstitial syndrome | 3 (0,1%) | 0 (0%) | 3 (0%) | 1 (1) |
| other | 87 (6%) | 4 (11%) | 66 (4%) | 0,10 (1) |
| Patients with multiple respiratory diseases; N (%) | 20 (1%) | 1 (3%) | 19 (1%) | 0,39 (1) |

1. Pearson's Chi-squared test. 2. Fisher's Exact Test for Count Data. 3 Wilcoxon test. COPD: Chronic Obstructive Pulmonary Disease. N: number of patients. RILI+: Radio-Induced Lung Injury presenting patients. RILI-: Radio-Induced Lung Injury negative patients.
