# Supplementary material for: Radiation-induced lung injury after breast cancer treatment: incidence in the CANTO-RT cohort and associated clinical and dosimetric risk factors
Source: Front Oncol. 2023 Jun 29;13:1199043. doi: 10.3389/fonc.2023.1199043 (PMC10342531; doi:10.3389/fonc.2023.1199043)
Supplement: Supplementary file 3 [file Table_3.docx]

**Table S3: Detailed tumor characteristics at inclusion**

|  | Overall (N=1565)  N (%) | RILI + (N=38)  N (%) | RILI - (N=1527)  N (%) | | p value |
| --- | --- | --- | --- | --- | --- |
| Histological type of right breast tumor |  |  |  | 0.49 (2) | |
| Ductal | 563 (82%) | 11 (79%) | 552 (82%) |  | |
| Lobular | 78 (11%) | 3 (21%) | 75 (11%) |  | |
| Mixt | 5 (1%) | 0 (0%) | 5 (1%) |  | |
| Other | 38 (6%) | 0 (0%) | 38 (6%) |  | |
| Missing | 881 | 24 | 857 |  | |
| Histological type of left breast tumor |  |  |  | 0.23 (2) | |
| Ductal | 635 (84%) | 15 (71%) | 620 (84%) |  | |
| Lobular | 85 (11%) | 4 (19%) | 81 (11%) |  | |
| Mixt | 5 (1%) | 0 (0%) | 5 (1%) |  | |
| Other | 30 (4%) | 2 (10%) | 28 (4%) |  | |
| Missing | 810 | 17 | 793 |  | |
| **Tumor Size (pT)*** |  |  |  | **< 0.01 (2)** | |
| 0 | 14 (1%) | 0 (0%) | 14 (1%) |  | |
| 1 | 1042 (67%) | 16 (42%) | 1026 (68%) |  | |
| 2 | 421 (27%) | 17 (45%) | 404 (27%) |  | |
| 3 | 78 (5%) | 5 (13%) | 73 (5%) |  | |
| Missing | 10 | 0 | 10 |  | |
| **Nodal Status (pN)*** |  |  |  | **< 0.01 (2)** | |
| 0 | 1035 (66%) | 16 (42%) | 1019 (67%) |  | |
| 1 | 408 (26%) | 14 (37%) | 394 (26%) |  | |
| 2 | 85 (5%) | 6 (16%) | 79 (5%) |  | |
| 3 | 35 (2%) | 2 (5%) | 33 (2%) |  | |
| Missing | 2 | 0 | 2 |  | |
| Grade |  |  |  | 0.07 (2) | |
| I | 266 (17%) | 2 (6%) | 264 (18%) |  | |
| II | 812 (53%) | 25 (69%) | 787 (52%) |  | |
| III | 464 (30%) | 9 (25%) | 455 (30%) |  | |
| Missing | 23 | 2 | 21 |  | |
| Laterality |  |  |  | 0.26 (2) | |
| Bilateral | 22 (1%) | 1 (3%) | 21 (1%) |  | |
| Left | 810 (52%) | 23 (61%) | 787 (52%) |  | |
| Right | 733 (47%) | 14 (37%) | 719 (47%) |  | |
| Missing | 0 | 0 | 0 |  | |
| RH + HER2 - |  |  |  | 0.34 (1) | |
| No | 209 (13%) | 7 (18%) | 202 (13%) |  | |
| Yes | 1342 (87%) | 31 (82%) | 1311 (87%) |  | |
| Missing | 14 | 0 | 14 |  | |
| HER2 + |  |  |  | 0.07 (1) | |
| No | 1333 (95%) | 29 (88%) | 1304 (95%) |  | |
| Yes | 68 (5%) | 4 (12%) | 64 (5%) |  | |
| Missing | 164 | 5 | 159 |  | |
| Triple Negative |  |  |  | 1.00 (1) | |
| No | 1304 (90%) | 32 (91%) | 1272 (90%) |  | |
| Yes | 141 (10%) | 3 (9%) | 138 (10%) |  | |
| Missing | 120 | 3 | 117 |  | |
| Ki67 (% of positive cells) |  |  |  | 0.21 (3) | |
| Median (Range) | 15.0 (0.0, 100.0) | 20.0 (2.0, 50.0) | 15.0 (0.0, 100.0) |  | |
| Missing | 1011 | 25 | 986 |  | |

1. Pearson's Chi-squared test. 2. Fisher's Exact Test for Count Data. 3 Wilcoxon test. RILI+: Radio-Induced Lung Injury presenting patients. RILI-: Radio-Induced Lung Injury negative patients. * according to TNM 7 (2010) version
