# Supplementary material for: Radiation-induced lung injury after breast cancer treatment: incidence in the CANTO-RT cohort and associated clinical and dosimetric risk factors
Source: Front Oncol. 2023 Jun 29;13:1199043. doi: 10.3389/fonc.2023.1199043 (PMC10342531; doi:10.3389/fonc.2023.1199043)
Supplement: Supplementary file 4 [file Table_4.docx]

**Table S4: Detailed treatment characteristics**

|  | Overall  (N=1565) | RILI +  (N=38) | RILI - (N=1527) | p value |
| --- | --- | --- | --- | --- |
| Treatment Site; N (%) |  |  |  | 0.53 (1) |
| Center N1 | 659 (42%) | 19 (50%) | 640 (42%) |  |
| Center N2 | 614 (39%) | 14 (37%) | 600 (39%) |  |
| Center N3 | 292 (19%) | 5 (13%) | 287 (19%) |  |
| Missing | 0 | 0 | 0 |  |
| **Type of Surgery; N (%)** |  |  |  | **0.02 (2)** |
| Tumorectomy | 1243 (79%) | 25 (66%) | 1218 (80%) |  |
| Mastectomy | 310 (20%) | 12 (32%) | 298 (20%) |  |
| Bilateral Mastectomy | 3 (0%) | 0 (0%) | 3 (0%) |  |
| Bilateral Tumorectomy | 7 (0%) | 0 (0%) | 7 (0%) |  |
| Tumorectomy and Mastectomy | 2 (0%) | 1 (3%) | 1 (0%) |  |
| Missing | 0 | 0 | 0 |  |
| **Chemotherapy; N (%)** |  |  |  | **< 0.01 (1)** |
| No | 724 (46%) | 9 (24%) | 715 (47%) |  |
| Yes | 839 (54%) | 29 (76%) | 810 (53%) |  |
| Missing | 2 | 0 | 2 |  |
| - Adjuvant chemotherapy |  |  |  | 0.56 (1) |
| No | 222 (26%) | 9 (31%) | 213 (26%) |  |
| Yes | 622 (74%) | 20 (69%) | 602 (74%) |  |
| Missing | 0 | 0 | 0 |  |
| - Neoadjuvant chemotherapy |  |  |  | 0.08 (1) |
| No | 1344 (86%) | 29 (76%) | 1315 (86%) |  |
| Yes | 219 (14%) | 9 (24%) | 210 (14%) |  |
| Missing | 2 | 0 | 2 |  |
| Associated hormonotherapy; N (%) |  |  |  | 0.96 (1) |
| No | 293 (19%) | 7 (18%) | 286 (19%) |  |
| Yes | 1269 (81%) | 31 (82%) | 1238 (81%) |  |
| Missing | 3 | 0 | 3 |  |
| - Type of associated hormonotherapy; N (%) |  |  |  | 0.88 (1) |
| Aromatase Inhibitor | 712 (56%) | 17 (55%) | 695 (56%) |  |
| Tamoxifen | 555 (44%) | 14 (45%) | 541 (44%) |  |
| Missing | 2 | 0 | 2 |  |
| Associated Trastuzumab; N (%) |  |  |  | 0.26 (1) |
| No | 1368 (88%) | 31 (82%) | 1337 (88%) |  |
| Yes | 195 (12%) | 7 (18%) | 188 (12%) |  |
| Missing | 2 | 0 | 2 |  |
| **Nodal Area Radiotherapy; N (%)** |  |  |  | **< 0.01 (1)** |
| No | 1004 (64%) | 15 (39%) | 989 (65%) |  |
| Yes | 561 (36%) | 23 (61%) | 538 (35%) |  |
| Missing | 0 | 0 | 0 |  |
| **Internal Mammary Chain Radiotherapy; N (%)** |  |  |  | **< 0.01 (1)** |
| No | 1085 (69%) | 17 (45%) | 1068 (70%) |  |
| Yes | 480 (31%) | 21 (55%) | 459 (30%) |  |
| Missing | 0 | 0 | 0 |  |
| Radiotherapy Boost; N (%) |  |  |  | 0.57 (1) |
| No | 605 (39%) | 13 (34%) | 592 (39%) |  |
| Yes | 960 (61%) | 25 (66%) | 935 (61%) |  |
| Missing | 0 | 0 | 0 |  |
| Radiotherapy technic; N (%) |  |  |  | 0.40 (2) |
| Conformational 3D | 1509 (96%) | 36 (95%) | 1473 (96%) |  |
| IMRT | 56 (4%) | 2 (5%) | 54 (4%) |  |
| Missing | 0 | 0 | 0 |  |
| Fractionnation Scheme; N (%) |  |  |  | 0.32 (1) |
| Hypofractionnated | 89 (11%) | 1 (4%) | 88 (11%) |  |
| NormoFractionnated | 751 (89%) | 22 (96%) | 729 (89%) |  |
| Missing | 725 | 15 | 710 |  |

1. Pearson's Chi-squared test 2. Fisher's Exact Test for Count Data, 3 Wilcoxon test,

Vx Gy: % of ipsilateral lung volume receiving x Gy. Dmean: mean dose to the ipsilateral lung (Gy), IMRT: Intensity-modulated radiation therapy
