# Supplementary material for: Radiation-induced lung injury after breast cancer treatment: incidence in the CANTO-RT cohort and associated clinical and dosimetric risk factors
Source: Front Oncol. 2023 Jun 29;13:1199043. doi: 10.3389/fonc.2023.1199043 (PMC10342531; doi:10.3389/fonc.2023.1199043)
Supplement: Supplementary file 5 [file Table_5.docx]

**Table S5: Chemotherapy type characteristics among chemotherapy receiving patients**

|  | **Overall**  **(N=839)** | **RILI +**  **(N=29)** | **RILI -**  **(N=810)** | **P value** |
| --- | --- | --- | --- | --- |
| Taxol-containing chemotherapy |  |  |  | 0.51 (1) |
| - No | 20 (2%) | 1 (3%) | 19 (2%) |  |
| - Yes | 819 (98%) | 28 (97%) | 791 (98%) |  |
| Epirubicine-containing chemotherapy |  |  |  | 0.36 (1) |
| - No | 93 (11%) | 5 (17%) | 88 (11%) |  |
| - Yes | 746 (89%) | 24 (83%) | 722 (89%) |  |
| Cyclophosphamide-containing chemotherapy |  |  |  | 0.19 (1) |
| - No | 23 (3%) | 2 (7%) | 21 (3%) |  |
| - Yes | 816 (97%) | 27 (93%) | 789 (97%) |  |

1Fisher's Exact Test for Count Data
