# Supplementary material for: Radiation-induced lung injury after breast cancer treatment: incidence in the CANTO-RT cohort and associated clinical and dosimetric risk factors
Source: Front Oncol. 2023 Jun 29;13:1199043. doi: 10.3389/fonc.2023.1199043 (PMC10342531; doi:10.3389/fonc.2023.1199043)
Supplement: Supplementary file 6 [file Table_6.docx]

**Table S6: Univariable analysis of RILI occurrence (logistic regression)**

| **Variables** | **n/N** | **OR** | **95% CI** | **P values** |
| --- | --- | --- | --- | --- |
| **Respiratory disease medical record** | **11/205** | **2.81** | **(1.37 , 5.78)** | **0.005** |
| **Cardiovascular disease medical record** | **5/449** | **0.37** | **(0.14 , 0.94)** | **0.038** |
| **pT** | **16/1042** | **2.26** | **(1.44 , 3.54)** | **<0.001** |
| **pN** | **14/407** | **1.84** | **(1.31 , 2.6)** | **<0.001** |
| **Associated chemotherapy** | **29/839** | **2.84** | **(1.34 , 6.05)** | **0.007** |
| Mastectomy | 12/310 | 1.9 | [0.95 , 3.82] | 0.07 |
| **Nodal area treatment** | **23/561** | **2.82** | **(1.46 , 5.45)** | **0.002** |
| **IMC treatment** | **21/480** | **2.87** | **(1.50 , 5.50)** | **0.001** |
| **V 5 Gy** |  | **1.02** | **(1.01 , 1.03)** | **0.002** |
| **V 10 Gy** |  | **1.02** | **(1.01 , 1.04)** | **0.008** |
| **V 15 Gy** |  | **1.03** | **(1.01 , 1.06)** | **0.003** |
| **V 20 Gy** |  | **1.05** | **(1.02 , 1.07)** | **<0.001** |
| **V 25 Gy** |  | **1.06** | **(1.03 , 1.09)** | **<0.001** |
| **V 30 Gy** |  | **1.08** | **(1.04 , 1.12)** | **<0.001** |
| **V 35 Gy** |  | **1.1** | **(1.05 , 1.15)** | **<0.001** |
| **V 40 Gy**  **Dmean** |  | **1.09**  **1.09** | **(1.03 , 1.15)**  **(1.03 , 1.15)** | **0.002**  **0.002** |

n/N: number of RILI / number of patients; OR: Odds Ratio CI: Confidence Interval; RT: Radiotherapy; Vx Gy: % of ipsilateral lung volume receiving x Gy. Dmean: mean dose to the ipsilateral lung (Gy), IMC: internal mammary chain
