# Supplementary material for: Radiation-induced lung injury after breast cancer treatment: incidence in the CANTO-RT cohort and associated clinical and dosimetric risk factors
Source: Front Oncol. 2023 Jun 29;13:1199043. doi: 10.3389/fonc.2023.1199043 (PMC10342531; doi:10.3389/fonc.2023.1199043)
Supplement: Supplementary file 7 [file Table_7.docx]

**Table S7: Contingency tables**

|  | V20>20% | V20≤20% |
| --- | --- | --- |
| No RILI | 458 | 1069 |
| RILI | 21 | 17 |

|  | V20>29% | V20≤29% |
| --- | --- | --- |
| No RILI | 149 | 1378 |
| RILI | 5 | 33 |

|  | V30>10% | V30≤10% |
| --- | --- | --- |
| No RILI | 705 | 822 |
| RILI | 29 | 9 |

| \|  \| V30>20% \| V30≤20% \| \| --- \| --- \| --- \| \| No RILI \| 188 \| 1339 \| \| RILI \| 10 \| 28 \|  \|  \| Dmean>15Gy \| Dmean≤15Gy \| \| --- \| --- \| --- \| \| No RILI \| 121 \| 1406 \| \| RILI \| 9 \| 29 \|  \|  \| Dmean>10Gy \| Dmean≤10Gy \| \| --- \| --- \| --- \| \| No RILI \| 520 \| 1007 \| \| RILI \| 24 \| 14 \| |
| --- | --- | --- | --- | --- | --- | --- | --- | --- | --- | --- | --- | --- | --- | --- | --- | --- | --- | --- | --- | --- | --- | --- | --- | --- | --- | --- | --- |
|  |
|  |
