# Supplementary material for: Radiation-induced lung injury after breast cancer treatment: incidence in the CANTO-RT cohort and associated clinical and dosimetric risk factors
Source: Front Oncol. 2023 Jun 29;13:1199043. doi: 10.3389/fonc.2023.1199043 (PMC10342531; doi:10.3389/fonc.2023.1199043)
Supplement: Supplementary file 8 [file Table_8.docx]

**Table S8: Positive and negative predictive values of different formerly published thresholds**

| **Parameter** | **PPV** | **NPV** | **PPV/NPV** | **OR*** | **p-value*** |
| --- | --- | --- | --- | --- | --- |
| **V20 Gy > 20%^8, 9, 11^** | **0.044** | **0.984** | **0.045** | **10.0** | **0.002** |
| V20 Gy > 29%^9^ | 0.032 | 0.977 | 0.033 | 0.2 | 0.67 |
| **V25 Gy > 10%^12^** | **0.035** | **0.988** | **0.035** | **7.1** | **0.007** |
| **V30 Gy > 10 %^13^** | **0.040** | **0.989** | **0.040** | **12.3** | **0.0004** |
| **V30 Gy > 20%^14^** | **0.051** | **0.980** | **0.051** | **5.4** | **0.02** |
| **Dmean > 10 Gy^9^** | **0.044** | **0.986** | **0.045** | **12.6** | **0.0004** |
| **Dmean > 15 Gy^14^** | **0.069** | **0.980** | **0.070** | **10.1** | **0.001** |

PPV: Predictive Positive Value; NPV: Negative Predictive Value; OR: Odds Ratio; * using a Chi2 test; Vx Gy: % of ipsilateral lung volume receiving x Gy; Dmean: mean dose to the ipsilateral lung (Gy);

^x^: reference for the published threshold

**Bibliography:**

8. Lind PARM, Wennberg B, Gagliardi G, Fornander T. Pulmonary complications following different radiotherapy techniques for breast cancer, and the association to irradiated lung volume and dose. Breast Cancer Res Treat. 1 août 2001;68(3):199‑210.

9. Zhou ZR, Han Q, Liang SX, He XD, Cao NY, Zi YJ. Dosimetric factors and Lyman normal-tissue complication modelling analysis for predicting radiation-induced lung injury in postoperative breast cancer radiotherapy: a prospective study. Oncotarget. 28 oct 2016;8(20):33855‑63.

11. Wen G, Tan YT, Lan XW, He ZC, Huang JH, Shi JT, et al. New Clinical Features and Dosimetric Predictor Identification for Symptomatic Radiation Pneumonitis after Tangential Irradiation in Breast Cancer Patients. J Cancer. 17 oct 2017;8(18):3795‑802.

12. Krengli M, Sacco M, Loi G, Masini L, Ferrante D, Gambaro G, et al. Pulmonary Changes After Radiotherapy for Conservative Treatment of Breast Cancer: A Prospective Study. International Journal of Radiation Oncology*Biology*Physics. 1 avr 2008;70(5):1460‑7.

13. Lee BM, Chang JS, Kim SY, Keum KC, Suh CO, Kim YB. Hypofractionated Radiotherapy Dose Scheme and Application of New Techniques Are Associated to a Lower Incidence of Radiation Pneumonitis in Breast Cancer Patients. Frontiers in Oncology [Internet]. 2020 [cité 21 avr 2022];10. Disponible sur: https://www.frontiersin.org/article/10.3389/fonc.2020.00124

14. Gokula K, Earnest A, Wong LC. Meta-analysis of incidence of early lung toxicity in 3-dimensional conformal irradiation of breast carcinomas. Radiat Oncol. 14 nov 2013;8(1):268.
