# Supplementary material for: Radiation-induced lung injury after breast cancer treatment: incidence in the CANTO-RT cohort and associated clinical and dosimetric risk factors
Source: Front Oncol. 2023 Jun 29;13:1199043. doi: 10.3389/fonc.2023.1199043 (PMC10342531; doi:10.3389/fonc.2023.1199043)
Supplement: Supplementary file 9 [file Table_9.docx]

**Table S9: Correlation coefficients between ipsilateral lung Vx Gy and mean dose**

|  | V5 Gy | V10 Gy | V15 Gy | V20 Gy | V25 Gy | V30 Gy | V35 Gy | V40 Gy | Dmean |
| --- | --- | --- | --- | --- | --- | --- | --- | --- | --- |
| V5 Gy | 1 | 0.98 | 0.96 | 0.94 | 0.92 | 0.88 | 0.80 | 0.64 | 0.96 |
| V10 Gy |  | 1 | 0.99 | 0.98 | 0.95 | 0.91 | 0.84 | 0.67 | 0.97 |
| V15 Gy |  |  | 1 | 0.99 | 0.97 | 0.94 | 0.87 | 0.70 | 0.98 |
| V20 Gy |  |  |  | 1 | 0.99 | 0.97 | 0.92 | 0.75 | 0.98 |
| V25 Gy |  |  |  |  | 1 | 0.98 | 0.95 | 0.79 | 0.98 |
| V30 Gy |  |  |  |  |  | 1 | 0.98 | 0.84 | 0.95 |
| V35 Gy |  |  |  |  |  |  | 1 | 0.91 | 0.91 |
| V40 Gy |  |  |  |  |  |  |  | 1 | 0.77 |
| Dmean |  |  |  |  |  |  |  |  | 1 |

Vx Gy: % of ipsilateral lung volume receiving x Gy. Dmean: mean dose to the ipsilateral lung (Gy).

Parameters presenting a correlation coefficient > 0.9 are highlighted in grey.
