# Supplementary material for: Radiation-induced lung injury after breast cancer treatment: incidence in the CANTO-RT cohort and associated clinical and dosimetric risk factors
Source: Front Oncol. 2023 Jun 29;13:1199043. doi: 10.3389/fonc.2023.1199043 (PMC10342531; doi:10.3389/fonc.2023.1199043)
Supplement: Supplementary file 10 [file Table_10.docx]

**Table S10: Dose-volume parameters thresholds from multivariable analyses (Logistic Regression)**

| **Variables** | **n/N** | | **OR** | | **95% CI** | **P values** | **AIC** | **BIC** |
| --- | --- | --- | --- | --- | --- | --- | --- | --- |
| Dmean > 5 Gy | | 25/822 | | 1.16 | [0.56 , 2.42] | 0.69 | 338 | 364 |
| **Dmean > 10 Gy** | | **15/262** | | **2.26** | **[1.11 , 4.62]** | **0.03** | **333** | **360** |
| Dmean > 15 Gy | | 3/30 | | 2.95 | [0.65 , 13.41] | 0.16 | 336 | 362 |
| Dmean > 20 Gy | | 0/4 | | 0 | [0 , inf] | 0.99 | 338 | 364 |
| V5 Gy > 5 % | | 34/1339 | | 0.78 | [0.25 , 2.45] | 0.67 | 338 | 364 |
| V5 Gy > 10 % | | 34/1273 | | 1.12 | [0.35 , 3.56] | 0.84 | 337 | 364 |
| V5 Gy > 15 % | | 32/1112 | | 1.19 | [0.42 , 3.35] | 0.74 | 338 | 364 |
| V5 Gy > 20 % | | 29/868 | | 1.34 | [0.48 , 3.74] | 0.58 | 338 | 364 |
| V5 Gy > 25 % | | 27/680 | | 1.99 | [0.62 , 6.40] | 0.25 | 337 | 363 |
| V5 Gy > 30 % | | 23/576 | | 1.28 | [0.24 , 6.67] | 0.77 | 338 | 364 |
| V5 Gy > 35 % | | 21/544 | | 0.64 | [0.16 , 2.47] | 0.52 | 337 | 364 |
| V5 Gy > 40 % | | 20/499 | | 0.91 | [0.28 , 2.92] | 0.88 | 338 | 364 |
| V10 Gy > 5 % | | 34/1293 | | 1.02 | [0.32 , 3.21] | 0.98 | 337 | 364 |
| V10 Gy > 10 % | | 34/1109 | | 2.21 | [0.70 , 7.00] | 0.18 | 336 | 362 |
| V10 Gy > 15 % | | 29/795 | | 1.80 | [0.64 , 5.09] | 0.27 | 336 | 363 |
| V10 Gy > 20 % | | 25/599 | | 1.71 | [0.40 , 7.28] | 0.47 | 337 | 364 |
| V10 Gy > 25 % | | 22/536 | | 1.32 | [0.30 , 5.72] | 0.71 | 338 | 364 |
| V10 Gy > 30 % | | 19/466 | | 1.08 | [0.38 , 3.04] | 0.89 | 338 | 364 |
| V10 Gy > 35 % | | 14/351 | | 1.01 | [0.43 , 2.34] | 0.99 | 338 | 364 |
| V10 Gy > 40 % | | 8/247 | | 0.76 | [0.32 , 1.81] | 0.53 | 337 | 364 |
| V15 Gy > 5 % | | 34/1265 | | 1.19 | [0.38 , 3.75] | 0.77 | 338 | 364 |
| V15 Gy > 10 % | | 30/975 | | 1.15 | [0.42 , 3.14] | 0.79 | 338 | 364 |
| V15 Gy > 15 % | | 28/665 | | 3.00 | [0.98 , 9.13] | 0.05 | 334 | 361 |
| V15 Gy > 20 % | | 24/541 | | 2.42 | [0.55 , 10.71] | 0.25 | 336 | 363 |
| V15 Gy > 25 % | | 18/446 | | 1.10 | [0.42 , 2.89] | 0.85 | 338 | 364 |
| V15 Gy > 30 % | | 11/297 | | 0.90 | [0.39 , 2.06] | 0.80 | 338 | 364 |
| V15 Gy > 35 % | | 7/148 | | 1.45 | [0.58 , 3.61] | 0.43 | 337 | 363 |
| V15 Gy > 40 % | | 4/73 | | 1.71 | [0.55 , 5.25] | 0.35 | 337 | 363 |
| V20 Gy > 5 % | | 34/1236 | | 1.32 | [0.42 , 4.15] | 0.64 | 338 | 364 |
| V20 Gy > 10 % | | 30/908 | | 1.46 | [0.53 , 4.02] | 0.47 | 337 | 363 |
| **V20 Gy > 15 %** | | **27/611** | | **3.56** | **[1.12 , 11.37]** | **0.03** | **333** | **360** |
| V20 Gy > 20 % | | 21/479 | | 1.95 | [058 , 6.60] | 0.28 | 336 | 363 |
| V20 Gy > 25 % | | 14/316 | | 1.26 | [0.54 , 2.96] | 0.59 | 338 | 364 |
| V20 Gy > 30 % | | 5/117 | | 1.20 | [0.43 , 3.33] | 0.73 | 338 | 364 |
| V20 Gy > 35 % | | 4/47 | | 2.84 | [0.91 , 8.91] | 0.07 | 335 | 361 |
| V20 Gy > 40 % | | 0/21 | | 0 | [0 , inf] | 1 | 336 | 362 |
| V25 Gy > 5 % | | 34/1203 | | 1.49 | [0.47 , 4.69] | 0.50 | 337 | 364 |
| V25 Gy > 10 % | | 29/840 | | 1.48 | [0.54 , 4.10] | 0.45 | 337 | 364 |
| **V25 Gy > 15 %** | | **25/537** | | **3.36** | **[1.03 , 10.92]** | **0.04** | **334** | **360** |
| V25 Gy > 20 % | | 15/383 | | 0.98 | [0.41 , 2.35] | 0.97 | 338 | 364 |
| V25 Gy > 25 % | | 7/146 | | 1.36 | [0.54 , 3.39] | 0.51 | 337 | 364 |
| V25 Gy > 30 % | | 3/46 | | 1.90 | [0.53 , 6.74] | 0.32 | 337 | 364 |
| V25 Gy > 35 % | | 1/16 | | 1.81 | [0.22 , 14.59] | 0.58 | 337 | 364 |
| V25 Gy > 40 % | | 0/7 | | 0 | [0 , inf] | 1 | 337 | 364 |
| V30 Gy > 5 % | | 34/1141 | | 1.91 | [0.60 , 6.05] | 0.27 | 336 | 363 |
| V30 Gy > 10 % | | 29/734 | | 2.59 | [0.99 , 6.75] | 0.05 | 334 | 360 |
| **V30 Gy > 15 %** | | **23/457** | | **3.07** | **[1.04 , 9.05]** | **0.04** | **333** | **360** |
| V30 Gy > 20 % | | 10/198 | | 1.47 | [0.63 , 3.42] | 0.37 | 337 | 364 |
| V30 Gy > 25 % | | 3/45 | | 1.93 | [0.54 , 6.87] | 0.31 | 337 | 363 |
| V30 Gy > 30 % | | 1/16 | | 1.62 | [0.20 , 13.11] | 0.65 | 338 | 364 |
| V30 Gy > 35 % | | 0/5 | | 0 | [0 , inf] | 1 | 337 | 364 |
| V35 Gy > 5 % | | 33/1044 | | 2.11 | [0.74 , 6.05] | 0.16 | 336 | 362 |
| V35 Gy > 10 % | | 25/585 | | 2.15 | [0.92 , 5.01] | 0.08 | 335 | 361 |
| V35 Gy > 15 % | | 14/239 | | 1.94 | [0.87 , 4.43] | 0.11 | 335 | 361 |
| V35 Gy > 20 % | | 2/40 | | 1.37 | [0.31, 6.15] | 0.68 | 338 | 364 |
| **V35 Gy > 25 %** | | **2/11** | | **6.45** | **[1.25 , 33.17]** | **0.03** | **334** | **360** |
| V35 Gy > 30 % | | 0/1 | | 0 | [0 , Inf] | 0.99 | 338 | 364 |
| V40 Gy > 5 % | | 25/822 | | 1.16 | [0.56 , 2.42] | 0.69 | 338 | 364 |
| **V40 Gy > 10 %** | | **15/262** | | **2.26** | **[1.11 , 4.62]** | **0.03** | **333** | **360** |
| V40 Gy > 15 % | | 3/30 | | 2.95 | [0.65, 13.41] | 0.16 | 336 | 363 |
| V40 Gy > 20 % | | 0/4 | | 0 | [0 , inf] | 1 | 338 | 364 |

n/N: number of RILI / number of patients; OR: Odds Ratio adjusted for **baseline respiratory disease, chemotherapy, and nodal RT**; CI: Confidence Interval; RT: Radiotherapy; AIC: Akaike information criterion; BIC: Bayesian information criterion. Vx Gy: % of ipsilateral lung volume receiving x Gy. Dmean: mean dose to the ipsilateral lung (Gy). Inf: infinity.
