# Supplementary material for: Radiation-induced lung injury after breast cancer treatment: incidence in the CANTO-RT cohort and associated clinical and dosimetric risk factors
Source: Front Oncol. 2023 Jun 29;13:1199043. doi: 10.3389/fonc.2023.1199043 (PMC10342531; doi:10.3389/fonc.2023.1199043)
Supplement: Supplementary file 11 [file Table_11.docx]

**Table S11: Dose equivalence used and ROC curve AUC for results presented in Table S12**

| Dose in 2Gy/Fr | EQD2 when using 40 Gy/15 Fr | ROC AUC in EQD2 |
| --- | --- | --- |
| 5 Gy | 4.5 Gy | 0.65 |
| 20 Gy | 17 Gy | 0.66 |
| 30 Gy | 28 Gy | 0.68 |

ROC AUC : Area Under the ROC Curve; Fr: Fraction; EQD2: dose equivalent to a 2 Gy by fraction scheme; Vx Gy: % of ipsilateral lung volume receiving x Gy.
