# Supplementary material for: Radiation-induced lung injury after breast cancer treatment: incidence in the CANTO-RT cohort and associated clinical and dosimetric risk factors
Source: Front Oncol. 2023 Jun 29;13:1199043. doi: 10.3389/fonc.2023.1199043 (PMC10342531; doi:10.3389/fonc.2023.1199043)
Supplement: Supplementary file 12 [file Table_12.docx]

**Table S12: Multivariable analyses of EQD2 V30 Gy (Logistic Regression)**

| **Variables** | **n/N** | **OR** | **95% CI** | **P values** | **AIC** | **BIC** |
| --- | --- | --- | --- | --- | --- | --- |
| **Baseline respiratory disease** | **11/203** | **2.99** | **[1.48 , 6.38]** | **<0.01** |  |  |
| Chemotherapy | 29/837 | 1.72 | [0.72 , 4.11] | 0.22 |  |  |
| Nodal RT | 23/561 | 1.22 | [0.45 , 3.29] | 0.70 | 333 | 360 |
| **EQD2 V30 Gy *** |  | **1.06** | **[1.01 , 1.12]** | **0.04** |  |  |

* n=1558 patients with V30 Gy for 825 patients with normofractionated regimen and V28 Gy for 733 patients with hypofractionated regimen. n/N: number of RILI / number of patients; OR: Odds Ratio CI: Confidence Interval; RT: Radiation therapy; AIC: Akaike information criterion; BIC: Bayesian information criterion. Vx Gy: % of ipsilateral lung volume receiving x Gy. Dmean: mean dose to the ipsilateral lung (Gy); EQD2: dose equivalent to a 2 Gy by fraction scheme.
